# Human Vγ9Vδ2-T Cells Synergize CD4^+^ T Follicular Helper Cells to Produce Influenza Virus-Specific Antibody

**DOI:** 10.3389/fimmu.2018.00599

**Published:** 2018-04-04

**Authors:** Qingyun Chen, Kun Wen, Aizhen Lv, Ming Liu, Ke Ni, Zheng Xiang, Yinping Liu, Wenwei Tu

**Affiliations:** ^1^Department of Paediatrics and Adolescent Medicine, University of Hong Kong, Pokfulam, Hong Kong; ^2^State Key Laboratory of Oncology in Southern China, Sun Yat-Sen University Cancer Center, Guangzhou, China; ^3^State Key Laboratory of Respiratory Disease, Guangzhou Institute of Respiratory Disease, The First Affiliated Hospital of Guangzhou Medical University, Guangzhou Medical University, Guangzhou, China

**Keywords:** Vγ9Vδ2-T cell, T follicular helper cell, antigen-specific antibody, plasma cell, immunoglobulin class switching

## Abstract

Human Vγ9Vδ2-T cells recognize nonpeptidic antigens and exert effector functions against microorganisms and tumors, but little is known about their roles in humoral immune response against influenza virus infection. Herein, in the coculture of autologous human B cells, dendritic cells and/or naïve CD4 T cells, and Vγ9Vδ2-T cells, we demonstrated that Vγ9Vδ2-T cells could facilitate H9N2 influenza virus-specific IgG and IgM productions in a CD4 T cell-dependent manner. Vγ9Vδ2-T cells promoted the differentiation of CXCR5^+^PD1^+^CD4^+^ T follicular helper (Tfh) cells, CD19^+^IgD^−^CD38^++^ plasma cells (PCs), and drove B cell proliferation as well as immunoglobulin class switching. Interestingly, Vγ9Vδ2-T cells acquired Tfh-associated molecules such as CXCR5, PD1, CD40L, and ICOS during influenza virus stimulation, especially in the presence of CD4 T cells. Moreover, Vγ9Vδ2-T cells promoted CD4 T cells to secrete IL-13 and IL-21, and neutralizing IL-13 and IL-21 significantly reduced the number of CD19^+^IgD^−^CD38^++^ PCs. Using humanized mice, we further demonstrated that Vγ9Vδ2-T cells could synergize CD4 T cells to produce influenza virus-specific antibody. Our findings provide a greater scope for Vγ9Vδ2-T cells in adaptive immunity, especially for the Tfh development and humoral immune responses against influenza virus infection.

## Introduction

Influenza virus continues to threaten global human health with significant morbidity and mortality. Induction of influenza virus-specific neutralizing antibodies (Abs) by vaccines is the most efficient strategy, because the neutralizing Abs can conjugate virus particles at the first contact of their invasion and promote clearance by host cells (1, 2). To generate Abs with high affinity and pathogen-neutralization ability, naïve B cells are activated and divide in response to cognate antigen and subsequently undergo rounds of somatic hypermutation and selection (3). This process is tightly regulated by T follicular helper (Tfh) cells, which are the specialized subset of CD4 T cells providing help to B cells (4, 5). Tfh cells are characterized by expressing the surface molecules CXCR5, PD1, ICOS, and CD40 ligand (CD40L), the cytokine IL-21, and the transcription factor Bcl-6 that directs Tfh lineage commitment (6). These molecules are important for Tfh cell development, migration, and function (7). Tfh cell differentiation starts when naïve CD4^+^ T cells meet with dendritic cells (DCs), and these activated CD4^+^ T cells upregulate CXCR5 expression and become early precursors of Tfh cells. B cells are required as a second stage of antigen-presenting cells (APCs)-CD4^+^ T cell conjugation to maintain and complete Tfh differentiation. Fully polarized Tfh cells are located within germinal center (GC) and have high expression of CXCR5 and Bcl-6 (8, 9).

Vγ9Vδ2-T cells constitute 1–5% of circulating T cells in human peripheral blood and they can be expanded rapidly in response to microbes that produce the metabolite pyrophosphate (10, 11). Although Vγ9Vδ2-T cells only constitute a small proportion of human immune cells, their broad antiviral and antitumor activities have been well demonstrated (12, 13). Moreover, human Vγ9Vδ2-T cells are also capable of regulating adaptive immune response through antigen presentation or signal transduction by soluble factors or membrane-attached molecules (14, 15). However, the role of Vγ9Vδ2-T cells in the humoral response against influenza virus infection remains unknown.

In mice, production of class-switched Abs, especially IgG1 and IgE can be efficiently generated in TCRα^−/−^ mice. Well-developed GCs are also present in the secondary lymphoid organs of TCRα^−/−^ and TCRβ^−/−^ mice (16, 17), indicating that γδ T cells may participate in antibody production. More recently, the deficiency in certain γδ T cell subsets was found to alter antibody production and shape peripheral B cell populations in non-immunized mice (18, 19). However, γδ T cell-deficient mice did not show marked defects in antibody production, therefore, γδ T cells may have a modulatory, rather than a dominant role in the control of humoral immunity (20). In human, γδ T cells can be detected in secondary lymphoid tissues such as gastrointestinal lymph nodes and intestinal mucosa-associated follicles, and express costimulatory molecules after TCR triggering, suggesting a role of γδ T cells in the maintenance of humoral immune responses after microbial infections (21). Subsequent studies demonstrated that phosphoantigen and IL-21 significantly induced Vγ9Vδ2-T cells to express Tfh cell-associated molecules such as CXCR5, CD40L, ICOS, OX40, and CD70, and these Vγ9Vδ2-T cells could support B cells to produce large amounts of IgM, IgG, and IgA *in vitro* cultures (21, 22). Although Vγ9Vδ2-T cells have been shown to provide potent B cell help during *in vitro* antibody production, it is still unknown about their roles in influenza virus-specific antibody production, CD4^+^ Tfh cell differentiation, and the combinational role of Vγ9Vδ2-T cells and CD4^+^ Tfh cells in the production of influenza virus-specific antibody.

Plasma cells (PCs) and their precursors play pivotal roles in humoral immune response by secreting antigen-specific immunoglobulin (Ig) (3). In the GCs, B cells undergo an iterative process of proliferation, somatic mutation of their rearranged Ig genes before differentiating into PCs, and Ig isotype switching in B cells has been found to be linked to cell division (23, 24). Most aspects of PC differentiation can be effectively recapitulated *in vitro* in response to Tfh cell-derived stimuli, such as CD40 ligation and cytokines including IL-4, IL-5, IL-10, IL-6, and IL-21 (25–28). IL-21, IL-4, and IL-13 were demonstrated to promote B cell survival, proliferation, isotype switching, and differentiation into Ig-secreting PCs (29, 30). Although IL-13 is a less efficient promoter of B cell growth than IL-4, it can induce the isotype switching of CD40L-actived naïve B cells in a division-linked, time-independent manner (24, 31). While much is known about the CD4^+^ Tfh cell-induced PC differentiation, our understanding about the effect of Vγ9Vδ2-T cells on the PC differentiation and isotype switching during influenza virus infection is still limited.

The aim of our work is to examine the role of Vγ9Vδ2-T cells in antigen-specific antibody production, PC differentiation, as well as B cell Ig isotype switching during influenza virus stimulation, and then applied humanized mice to confirm their effects *in vivo*. We demonstrated that Vγ9Vδ2-T cells could increase H9N2 influenza virus-specific IgG and IgM productions in a CD4 T cell-dependent manner both *in vitro* and *in vivo*. During this process, Vγ9Vδ2-T cells also acquired Tfh-associated features especially in the presence of CD4 T cells, indicating a reciprocal effect between Vγ9Vδ2-T and CD4 T cells in the differentiation of Tfh-like cells.

## Materials and Methods

### Cells Preparation

Human CD14^+^ monocytes were isolated from human peripheral blood mononuclear cells (PBMC) using the anti-CD14^+^ microbeads (Miltenyi Biotec). The CD14^+^ monocytes were cultured with GM-CSF (50 ng/ml) and IL-4 (10 ng/ml) for 5–7 days to differentiate into immature DCs. 5 × 10^4^ immature DCs were seeded in 96-well U-bottom plate and were pulsed with UV-inactivated H9N2 influenza viruses (A/Quail/HK/G1/97, MOI = 5) and LPS (100 ng/ml). Naïve CD4^+^ T cells were isolated from autologous PBMC using the naïve CD4^+^ T cells isolation kit (Miltenyi Biotec). 2.5 × 10^5^ naïve CD4^+^ T cells in each well were cultured with the UV-H9N2 influenza viruses pulsed DCs in 96-well U-bottom plate for 5 days. On day 5 after naïve CD4 T cells and DCs being cocultured, human CD19^+^ B cells and Vγ9Vδ2-T cells were isolated from autologous PBMC using human CD19 microbeads (Miltenyi) and TCR γδ T cells isolation Kit (Miltenyi), respectively. 1 × 10^4^ DCs/well were seeded in 96 well U-bottom plate the day before Vγ9Vδ2-T cells and B cells isolation and were treated with UV-H9N2 (MOI = 5). 5 × 10^4^ CD4 T cells that have been cultured with UV-H9N2 pulsed DCs were added in 96 well U-bottom plate (with UV-H9N2 pulsed DCs) according to different groups. 1 × 10^5^ B cells were added in all groups and 1 × 10^5^ Vγ9Vδ2-T cells were added in 96 well U-bottom plate in indicated group. B cells were stimulated with UV-H9N2 at the MOI of 2. Cells were cultured in Iscove’s Modified Dulbecco’s Medium (Invitrogen), supplemented with 10% FBS, 50 µg/ml transferrin (Roche Diagnostics, Indianapolis, IN, USA), 5 µg/ml insulin (Sigma-Aldrich, St. Louis, MO, USA), and 15 µg/ml gentamicin (Invitrogen), 40 ng/ml human IL-4 (Peprotech). In all the coculture experiments, all cultured cells (CD14^+^ monocytes, naïve CD4^+^ T cells, CD19^+^ B cells, and Vγ9Vδ2-T cells) were isolated from the same donor. The cell purity was above 95% after magnetic cell sorting.

### Flow Cytometry

Following Abs were used for flow staining: anti-TCR Vδ2 (B6), anti-TCR Vγ9 (B1), anti-CD14 (63D3), anti-CD4 (RPA-T4), anti-CD19 (HIB19), anti-CXCR5 (J252D4), anti-PD1 (EH12.2H7), anti-Bcl-6 (7D1), anti-CD40 (5C3), anti-CD40L (24–31), anti-ICOS (C398.4A), anti-ICOSL (2D3), anti-OX40 (ACT35), anti-CD38 (HB-7), anti-IgD (LA6-2), anti-IgG (HP6017), anti-IgA (IS11-8E10), anti-Ki67 (Ki67), anti-IL-13 (JES10-5A2), and anti-IL-21 (4BG1). All Abs were purchased from BioLegend. For surface markers staining, cells pre-washed with PBS were incubated with specific Abs or their corresponding isotype control Abs for 15 min in the dark at room temperature. For intracellular staining, cells that stained with surface markers were fixed and permeabilized using the Lysing Solution (BD) and Permeabilizing Solution 2 (BD), respectively. Subsequently, the cells were incubated with specific Abs and their corresponding isotype control for 30 min in the dark at room temperature. After washing with PBS, the cells were analyzed by FACS LSR II and Flowjo software as mentioned before (13, 32).

### Flowcytomix Assay

To detect the cytokine production in supernatant, the bead-based flowcytomix assay was used according to the manual provided by the manufacturer (BioLegend, LEGENDplex™ USA). The concentration of the cytokines was calculated by extrapolating the mean fluorescence intensity on the respective standard curves and expressed as picogram per milliliter.

### Transwell Assay

1 × 10^4^ DCs/well were seeded at the bottom of 96 well-transwell plate (pore size: 0.3 μm, Millipore). 1 × 10^5^ CD19^+^ B cells and 5 × 10^4^ CD4 T cells that have been cultured with UV-H9N2 pulsed DCs were added directly to the bottom wells of the transwell plate. 1 × 10^5^ Vγ9Vδ2-T cells were added in the upper chamber or the lower chamber in indicated groups. In some inserts, CD4 T cells were separated from B cells in the upper chamber. The B cells in each group were pulsed with UV-H9N2 influenza viruses (MOI = 2). On the day 6–8 after incubation, the differentiation of PCs and CD4^+^ Tfh cells were determined by FACS LSR II.

### Cytokines Blocking Assay

For IL-13 blocking, IL-13 neutralizing antibody (1 µg/ml, BD Pharmingen™, USA) and IgG control antibody (1 µg/ml, BD Pharmingen™, USA) were used. For IL-21 blocking, recombinant human IL-21R Fc chimera protein (1 µg/ml, R&D systems) and recombinant human IgG1 Fc (1 µg/ml, R&D systems) were used. After 6 days’ culture with cells in different groups, the differentiation of CD4^+^ Tfh cells and PCs were determined by FACS LSRII.

### Enzyme-Linked Immunosorbent Assay (ELISA)

The amounts of total human IgG and IgM Abs in the supernatant were determined by human IgG and IgM ELISA Quantitation Set (Bethyl). For virus-specific IgG and IgM Abs production, 96 well plates were coated with UV-H9N2 influenza viruses in coating buffer (5 × 10^5^ virus/well) at 4°C overnight. Supernatant from different groups was added to the blocked plate and incubated for 2 h at 37°C. HRP conjugated human IgG and IgM detection antibody was added to each well for 1 h at 37°C. TMB substrate solution was added to each well. Fifteen minutes later, the reaction was stopped by adding 2 M hydrochloric acid (HCL). Plates were read at 450 nm.

### Influenza Hemagglutination Inhibition Assay (HAI Assay)

25 µl PBS was added in 96 well U-bottom plates from the second row to the last row. Supernatant or receptor destroyed enzyme-treated serum were added to the first row (50 μl/well) and serially diluted twofold. 4 HAU UV-inactivated H9N2 viruses in 25 µl volume PBS were added to each well. After 30 min incubation in 37°C, 0.05% turkey red blood cells were added to the plates and incubated for 40 min at room temperature. Then, the HAI titers were read and recorded.

### Immunohistochemistry

Samples of spleen from whole PBMC-humanized mice were fixed in 10% formalin, embedded in paraffin, sectioned, and stained with rabbit anti-human CD4 (EPR6855, Abcam), goat anti-human CD20 (Abcam), and mouse anti-human TCR γδ (B1, BioLegend) Abs. The immune localization of CD4 (Streptavidin Peroxidase, Red) and CD20 (Streptavidin Alkaline Phosphatase, Black), CD4 (Streptavidin Peroxidase, Red) and TCR γδ (Streptavidin Alkaline Phosphatase, Black), and CD20 (Streptavidin Peroxidase, Red) and TCR γδ (Streptavidin Alkaline Phosphatase, Black) were stained in spleen section using Immunohistochemistry Double Staining Kit (DouSPTM, MXB, China).

### Generation of Humanized Mice in Rag2^−/−^γc^−/−^ Immune Deficient Mice

Vγ9Vδ2-T cell-depleted PBMC, CD4 T cell-depleted PBMC, and both Vγ9Vδ2-T and CD4 T cell-depleted PBMC were obtained after depletion cells by magnetic microbeads (Miltenyi Biotec). 4- to 5-week-old male or female Rag2^−/−^γc^−/−^ mice were treated with liposomes (VU Medisch Centrum, Amsterdam, the Netherlands) 1 day before transplantation. The sublethally irradiated mice were transplanted i.p. with 30 × 10^6^ PBMC or specific cell-depleted PBMC. The immune reconstitution of humanized mice was according to published paper in our group (13, 32, 33). Four weeks later, established humanized mice in different groups were vaccinated UV-inactivated H9N2 influenza viruses (10^3^TCID_50_) through i.p. pathway on day 0 and 7. Serum was collected on day 21 postvaccination, and total IgG, IgM, and virus-specific Abs were determined by ELISA and HAI assay.

### Ethics Statement

Human PBMC were collected from the buffy coats, which were obtained from the Hong Kong Red Cross approved by the Institutional Review Board of the University of Hong Kong/Hospital Authority Hong Kong West Cluster (IRB number: UW 07-154). All animal studies were approved and performed in compliance with the guidelines for the use of experimental animals by the Committee on the Use of Live Animals in the Teaching and Research (CULATR), the University of Hong Kong (CULATR number: 2986-13).

### Statistical Analysis

Data were analyzed using paired Student’s *t*-tests or two-way ANOVA (GraphPad Prism 5.0), with differences considered significant as indicated in the figures: *p < 0.05; **p < 0.01; ***p < 0.001.

## Results

### Vγ9Vδ2-T Cells Facilitated Influenza Virus-Specific Antibody Production

**Figure 1:**
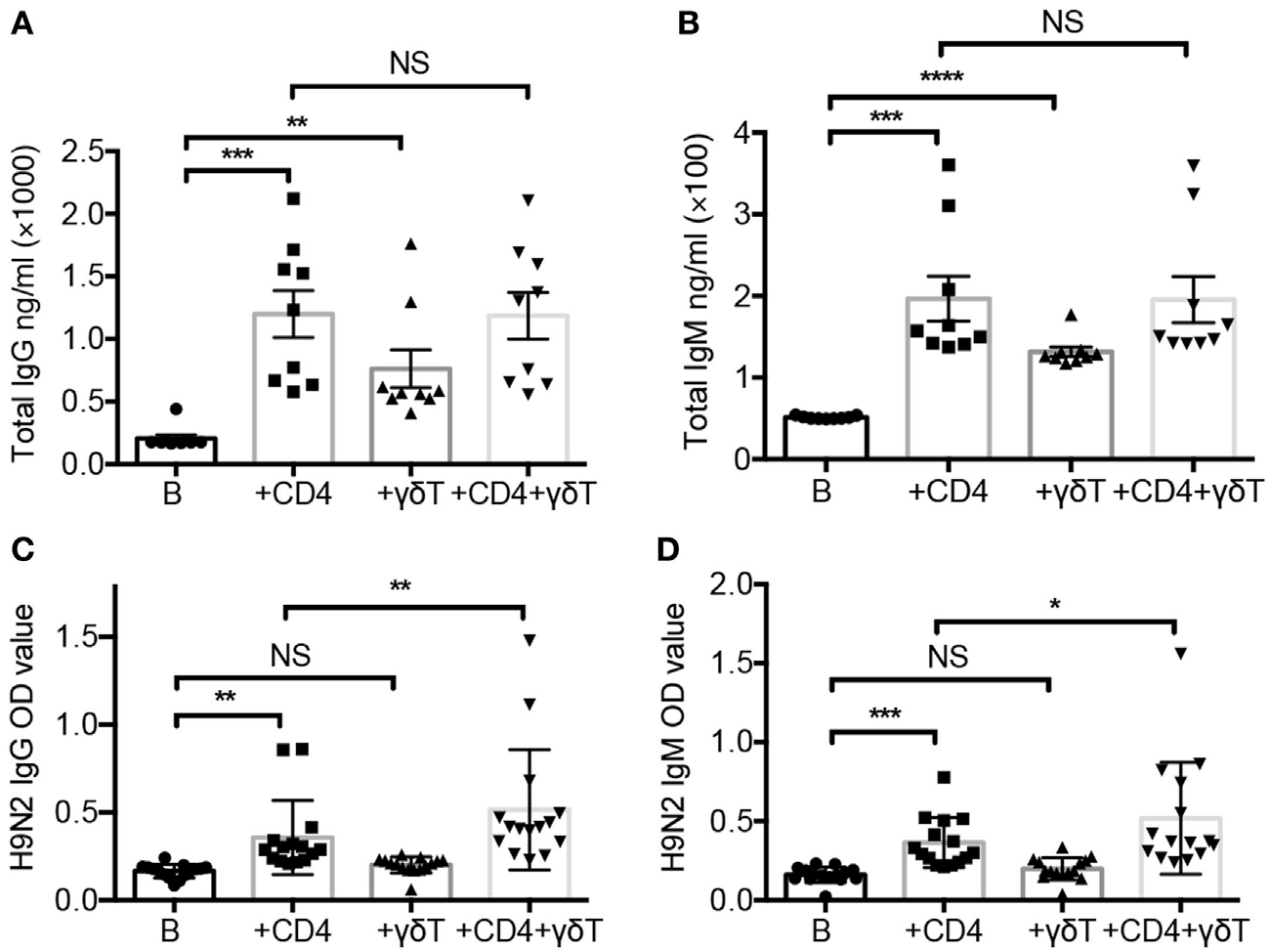
Vγ9Vδ2-T cells facilitated influenza virus-specific antibody production. Naïve CD4 T cells were cultured with UV-H9N2 virus-pulsed dendritic cells for 4–5 days, then, the activated CD4 T cells were cultured with CD19^+^ B cells from same donor with or without Vγ9Vδ2-T cells for another 7–10 days. **(A,B)** Total IgG and IgM in supernatant on day 7 were detected by enzyme-linked immunosorbent assay (ELISA). **(C,D)** H9N2 virus-specific IgG and IgM were detected by ELISA on day 7. Each dot means one donor. The data shown are the mean ± SEM. *p < 0.05; **p < 0.01; ***p < 0.001; ****p < 0.0001. NS, no significant difference.

To investigate the role of Vγ9Vδ2-T cells in antibody response against influenza virus, B cells were cocultured with CD4 and/or Vγ9Vδ2-T (γδ T) cells for 7–10 days in the presence of UV-H9N2-pulsed DCs, and the total and influenza virus-specific Abs in the supernatants were determined. As shown in Figures 1A,B, CD4 and Vγ9Vδ2-T cells helped B cells produce total IgM and IgG Abs, respectively, but the helper efficiency of Vγ9Vδ2-T cells was lower than that of CD4 T cells. Vγ9Vδ2-T cells could not further enhance the helper capacity of CD4 T cells for B cells to produce total IgM and IgG production. Interestingly, Vγ9Vδ2-T cells showed a synergizing effect with CD4 T cells on inducing H9N2 virus-specific IgG and IgM, although Vγ9Vδ2-T cells alone only had a little effect on virus-specific antibody production (Figures 1C,D). The numbers of B cells and CD4 T cells were shown in Figures S1A,B in Supplementary Material. To address if the Tfh-like Vγ9Vδ2-T cells could help B cells directly after activation by CD4 T cell, we culture the naïve CD4 T cells with fresh isolated Vγ9Vδ2-T cells, then, the pre-activated Vγ9Vδ2-T cells were isolated to stimulate B cells producing Abs. We found that the pre-activated Vγ9Vδ2-T alone could not promote H9N2 virus-specific antibody production even when they were cultured with CD4 T cells before (Figures S1C–F in Supplementary Material). The pre-activated Vγ9Vδ2-T cells did not show differences in cytokines secretion such as IL-13, IL-21, and IL-6 compared with fresh Vγ9Vδ2-T cells (Figures S1G–J in Supplementary Material). Our results demonstrated that Vγ9Vδ2-T cells were unable to improve virus-specific antibody productions by themselves, but they could induce total IgG and IgM productions and facilitate virus-specific antibody productions in a CD4 T cell-dependent manner.

### Vγ9Vδ2-T Cells Promoted CD4^+^ Tfh Cells Differentiation

**Figure 2:**
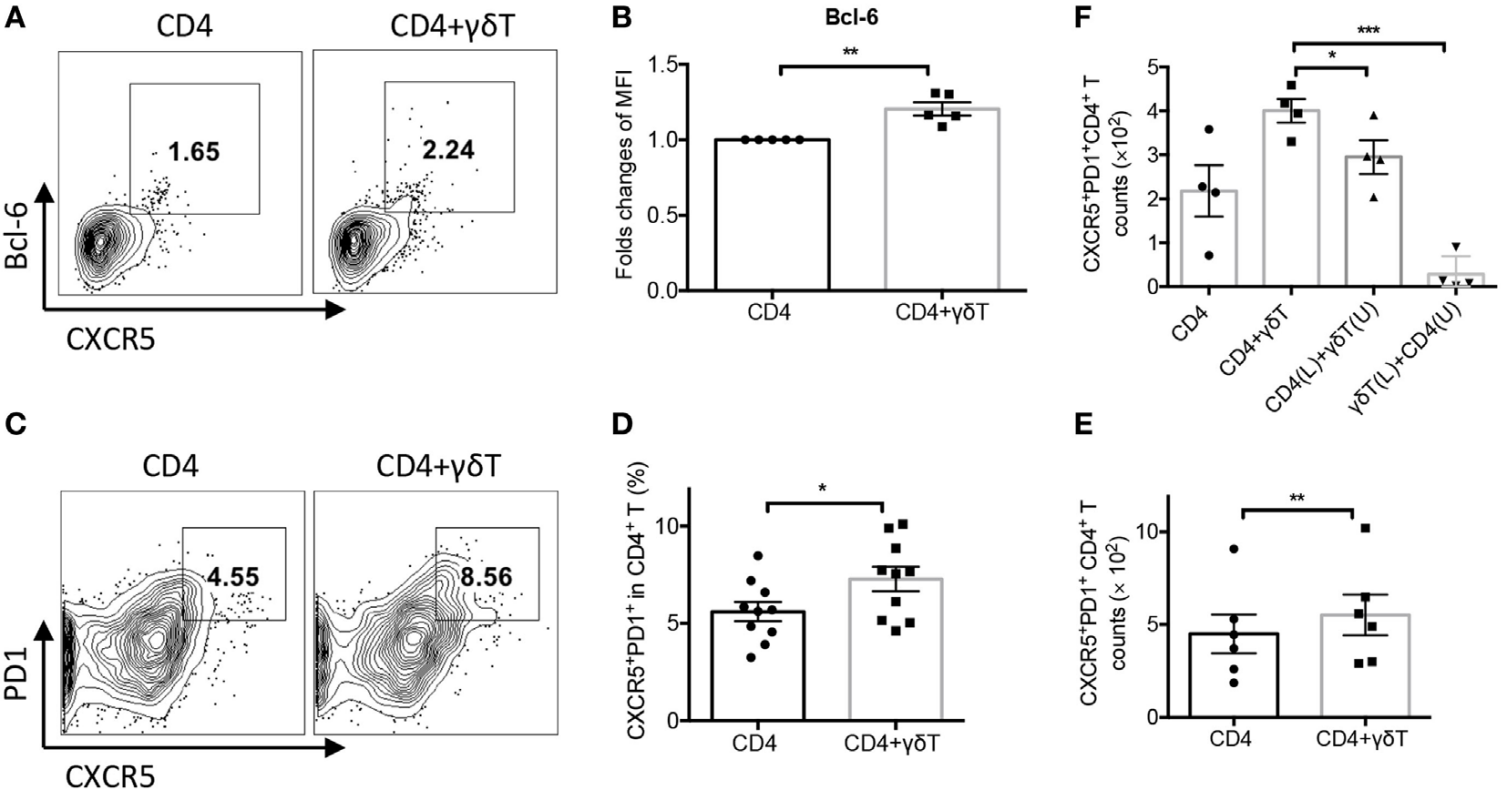
Vγ9Vδ2-T cells promoted CD4^+^ T follicular helper (Tfh) cells differentiation. Naïve CD4 T cells were cultured with UV-H9N2 virus-pulsed dendritic cells for 4–5 days, then, the activated CD4 T cells were cultured with CD19^+^ B cells from same donor with or without Vγ9Vδ2-T cells for another 7–10 days. **(A)** Percentage of CXCR5^+^Bcl-6^+^CD4^+^ Tfh cells was detected on day 5 by flow cytometry. **(B)** Bcl-6 mean fluorescence intensity (MFI) fold changes were detected on day 5 by flow cytometry. **(C–E)** Number and percentage of CXCR5^+^PD1^+^CD4^+^ Tfh cells were detected on day 5. **(F)** CD4^+^ T cells and Vγ9Vδ2-T cells were put in upper (U) or lower (L) chamber of transwell system in indicated group. Number of CXCR5^+^PD1^+^CD4^+^ Tfh cells were detected. FACS data represent four to nine independent experiments. Each dot means one donor. The data shown are the mean ± SEM. *p < 0.05; **p < 0.01; ***p < 0.001.

We further determined whether Vγ9Vδ2-T cells could facilitate CD4^+^ Tfh cell differentiation during influenza virus stimulation. As shown in Figures 2A,B, Vγ9Vδ2-T cells enhanced Bcl-6 expression in CD4 T cells. Vγ9Vδ2-T cells also increased the frequency and number of CXCR5^+^PD1^+^CD4^+^ Tfh cells (Figures 2C–E). These results demonstrated that Vγ9Vδ2-T cells could promote CXCR5^+^PD1^+^CD4^+^ Tfh cell differentiation. In addition, Vγ9Vδ2-T cells increased the number of CD40L^+^CD4^+^ T cells (Figure S2A in Supplementary Material), but they did not alter the expressions of OX40 and ICOS in CD4 T cells (Figures S2B,C in Supplementary Material). Using transwell system, we further found that the number of CXCR5^+^PD1^+^CD4^+^ Tfh cells significantly reduced when CD4- and Vγ9Vδ2-T cells were separated (Figure 2F). These results indicated that cell–cell contact was important for Vγ9Vδ2-T cells to provide “help” to improve CXCR5^+^PD1^+^CD4^+^ Tfh cell differentiation.

### CD4 T Cells Promoted Vγ9Vδ2 Tfh Cell Differentiation

**Figure 3:**
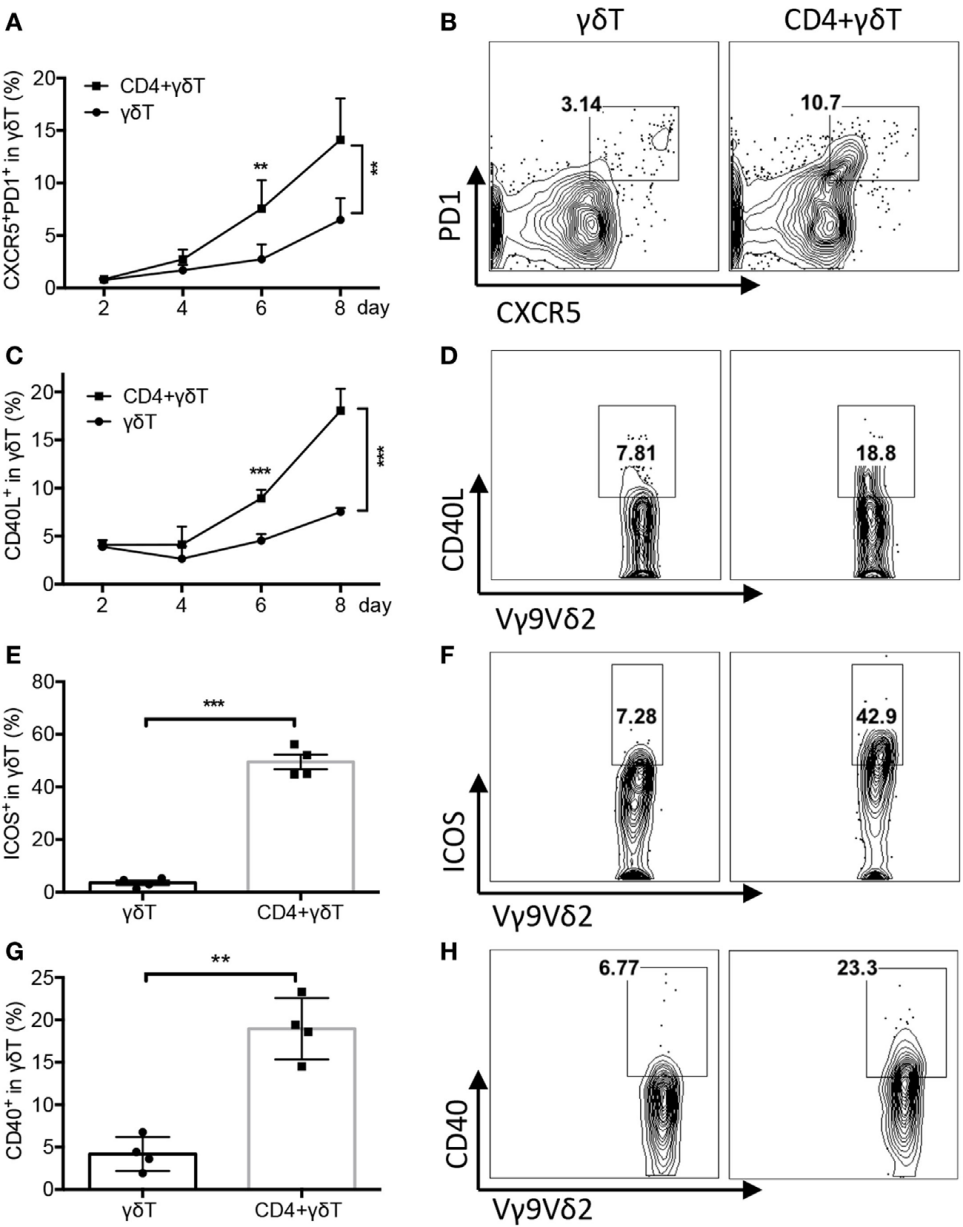
CD4 T cells promoted Vγ9Vδ2-T follicular helper cell differentiation. Naïve CD4 T cells were cultured with UV-H9N2 virus-pulsed dendritic cells for 4–5 days, then, the activated CD4 T cells were cultured with CD19^+^ B cells from same donor with or without Vγ9Vδ2-T cells for another 7–10 days. **(A–D)** Percentage of CXCR5^+^PD1^+^Vγ9Vδ2-T and CD40L^+^Vγ9Vδ2-T cells was detected by flow cytometry at different time points. FACS data showed the results of day 6. **(E–H)** Percentage of ICOS^+^ Vγ9Vδ2-T cells and CD40^+^ Vγ9Vδ2-T cells was detected on day 4. *n* = 4**(E)**, *n* = 3**(G)**. FACS data represent 3–10 independent experiments. The data shown are the mean ± SEM. **p < 0.01; ***p < 0.001.

To explore whether Vγ9Vδ2-T cells exhibit Tfh feature during the coculture, Tfh cell-associated molecules were examined. As shown in Figures 3A–D, Vγ9Vδ2-T cells acquired Tfh characteristics during the culture with B cells as evidenced by the increase of the frequency of Tfh-like CXCR5^+^PD1^+^ Vγ9Vδ2-T cells and CD40L^+^ Vγ9Vδ2-T cells. CD4 T cells further enhanced the frequency of Tfh-like CXCR5^+^PD1^+^ Vγ9Vδ2-T cells and CD40L^+^ Vγ9Vδ2-T cells during the coculture (Figures 3A–D). CD4 T cells also increased the expressions of ICOS and CD40 in Vγ9Vδ2-T cells (Figures 3E–H). These data demonstrated that Vγ9Vδ2-T cells acquired Tfh cell features after coculture with B cells, and CD4 T cells could further promote Vγ9Vδ2-T cells differentiation into Tfh cells.

### Vγ9Vδ2-T Cells Promoted PC Differentiation and B Cell Ig Class Switching

**Figure 4:**
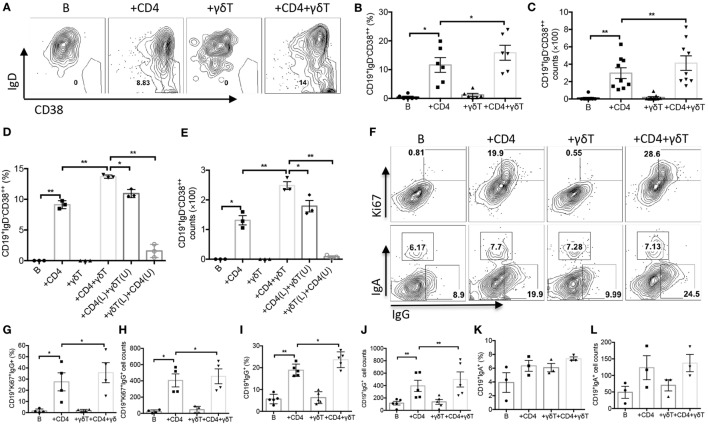
Vγ9Vδ2-T cells promoted plasma cell (PC) differentiation and B cell immunoglobulin class switching. Naïve CD4 T cells were cultured with UV-H9N2 virus-pulsed dendritic cells for 4–5 days, then, the activated CD4 T cells were cultured with CD19^+^ B cells from same donor with or without Vγ9Vδ2-T cells for another 7–10 days. **(A–C)** Percentage and number of CD19^+^IgD^−^CD38^++^ PCs were detected on day 6 by flow cytometry. FACS data represent eight independent experiments. **(D,E)** CD4 T cells and Vγ9Vδ2-T cells were put in upper (U) or lower (L) chamber of transwell system in indicated group. The percentage and number of CD19^+^IgD^−^CD38^++^ PCs were detected on day 6 by flow cytometry, *n* = 3. **(F–H)** The percentage and number of CD19^+^Ki67^+^IgG^+^ B cells were detected by flow cytometry on day 3. **(F,I–L)** The percentage and number of CD19^+^IgG^+^ and CD19^+^IgA^+^ B cells were detected by flow cytometry on day 3. FACS data represent four to five independent experiments. Each dot means one donor. The data shown are the mean ± SEM. *p < 0.05; **p < 0.01.

To investigate whether Vγ9Vδ2-T cells could promote PC differentiation, CD19^+^IgD^−^CD38^++^ PCs were examined. As shown in Figures 4A–C, Vγ9Vδ2-T cells alone did not support CD19^+^IgD^−^CD38^++^ PC differentiation, but they could synergize CD4 T cells to improve PC differentiation, as indicated by the increase of the frequency and number of CD19^+^IgD^−^CD38^++^ PCs during the coculture with both CD4- and Vγ9Vδ2-T cells compared to that with CD4 T cells alone. The direct contact of Vγ9Vδ2- and CD4 T cells was also required for the PC differentiation as the frequency and number of CD19^+^IgD^−^CD38^++^ PCs significantly reduced when these two cell populations were separated by transwell (Figures 4D,E). We also noted that PC differentiation was inefficient when CD4 T cells and B cells were separated (Figures 4D,E). These results indicated that PC differentiation was CD4 T cells dependent, and Vγ9Vδ2-T cells had a synergizing effect with CD4 T cells on promoting PC differentiation.

Before differentiating into PCs, activated B cells will undergo a strong proliferative burst, in which the proportion of cells that undergo isotype switching increases with division number (24, 34–38). We noted that the proportion and number of Ki67^+^IgG^+^CD19^+^ B cells significantly increased in the presence of both CD4 T and Vγ9Vδ2-T cells compared with CD4 T cells alone (Figures 4F,G). Almost all the IgG^+^ B cells also exhibited Ki67, indicating these B cells were undergoing cell division. However, we observed no change of isotype switching to IgA with or without Vγ9Vδ2-T cells (Figures 4F,K,L). Our data indicated that Vγ9Vδ2-T cells could improve the capacity of CD4 T cells to drive B cell proliferation and Ig class switching to IgG (Figures 4F–J).

### Vγ9Vδ2-T Cells Increased Productions of IL-13, IL-5, IL-6, and IL-21

**Figure 5:**
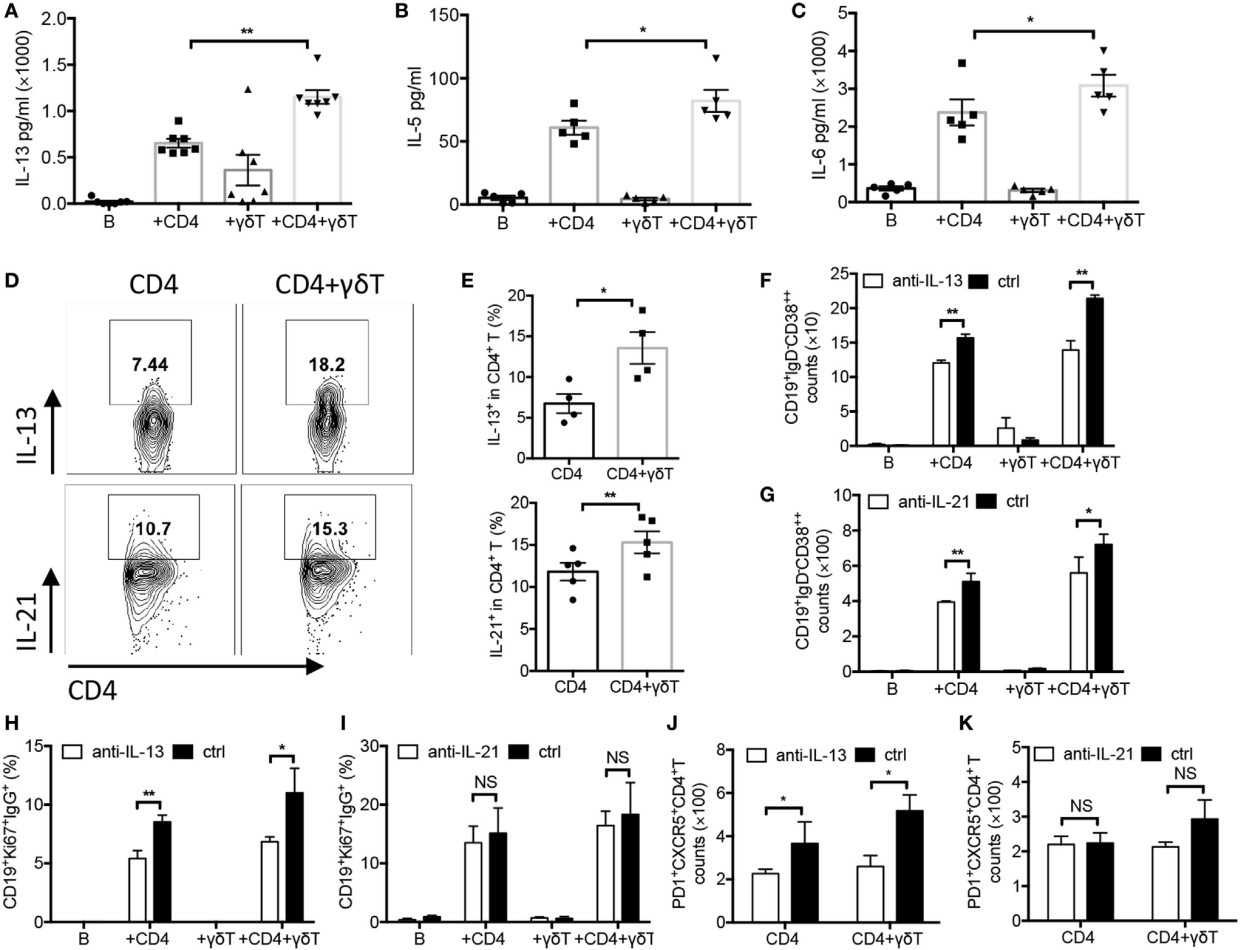
Vγ9Vδ2-T cells increased productions of IL-13, IL-5, IL-6, and IL-21. Naïve CD4 T cells were cultured with UV-H9N2 virus-pulsed dendritic cells for 4–5 days, then, the activated CD4 T cells were cultured with CD19^+^ B cells from same donor with or without Vγ9Vδ2-T cells for another 7–10 days. Supernatant in different groups were collected on day 5 and were detected by Cytometric Bead Array. Data showed the productions of IL-13 **(A)**, IL-5 **(B)**, and IL-6 **(C)**. **(D,E)** Intracellular IL-13 and IL-21 in CD4 T cells were detected on day 5 by stimulating cells with PMA, inomycin, and BFA within the last 6 h. FACS data represent four to six independent experiments. **(F–K)** IL-13 neutralizing antibody or IL-21 receptor Fc fusion protein and their controls were added into each group, the number of CD19^+^IgD^−^CD38^++^ plasma cells **(F,G)**, the frequency of CD19^+^Ki67^+^IgG^+^ B cells **(H,I)**, and the number of PD1^+^CXCR5^+^CD4^+^ T follicular helper cells **(J,K)** were detected on days 6, 3, and 5, respectively. Each dot means one donor. The data shown are the mean ± SEM. *p < 0.05; **p < 0.01. NS, no significant difference.

To investigate whether Vγ9Vδ2-T cells could induce cytokine release during PC differentiation, cytokine productions in supernatant were examined. We observed that Vγ9Vδ2-T cells significantly increased IL-13, IL-5, and IL-6, but not IL-10, TNF-α, or IL-12 productions in supernatant in the presence of CD4 T cells (Figures 5A–C; Figures S2D–F in Supplementary Material). By performing intracellular staining, higher IL-13- and IL-21-secreting CD4 T cells were observed after addition of Vγ9Vδ2-T cells (Figures 5D,E). However, no changes of IL-13- and IL-21-secreting Vγ9Vδ2-T cells were observed after addition of CD4 T cells (Figures S2G,H in Supplementary Material). These data demonstrated that Vγ9Vδ2-T cells could facilitate CD4 T cells to secrete IL-13 and IL-21. We further investigated the effects of IL-13 or IL-21 on PC differentiation. Blockade of IL-13 by its neutralization Abs significantly reduced the number of CD19^+^IgD^−^CD38^++^ PCs, the frequency of CD19^+^Ki67^+^IgG^+^ B cells, and the number of PD1^+^CXCR5^+^CD4^+^ Tfh cells (Figures 5F,H,J). Blockade of IL-21 signaling by IL-21R Fc chimera protein also decreased the number of CD19^+^IgD^−^CD38^++^ PCs (Figure 5G), but had no effect on the frequency of CD19^+^Ki67^+^IgG^+^ B cells and the number of PD1^+^CXCR5^+^CD4^+^ Tfh cells (Figures 5I,K). Anti-IL-13 and anti-IL-21 signaling Abs co-treatment showed similar effect in suppression PC differentiation compared with IL-13 or IL-21 neutralization antibody treatment alone (Figure S2J in Supplementary Material). These data indicated both IL-13 and IL-21 were involved in PC differentiation.

### Vγ9Vδ2-T Cells Increased H9N2 Virus-Specific IgG and IgM Productions *In Vivo*

**Figure 6:**
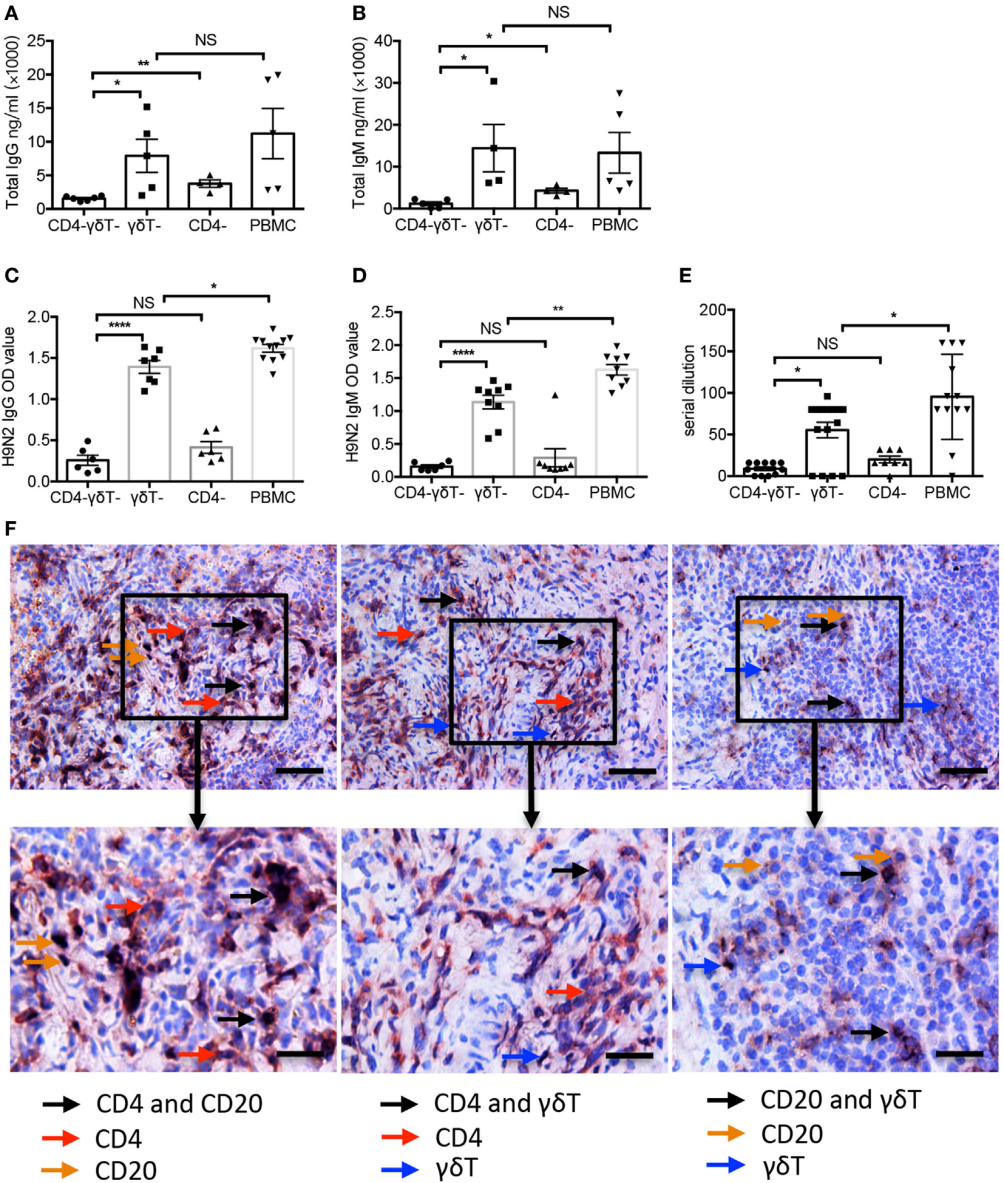
Vγ9Vδ2-T cells increased H9N2 virus-specific IgG and IgM productions *in vivo*. Specific immune cells-depleted and whole peripheral blood mononuclear cells (PBMC)-humanized mice were vaccinated with UV-inactivated H9N2 virus through i.p. pathway on day 0 and 7. Serum was collected on day 21 post the first vaccination. **(A,B)** Total IgG and IgM in serum **(C,D)** H9N2 virus-specific IgG and IgM in serum that treated with receptor destroyed enzyme (RDE) were determined by enzyme-linked immunosorbent assay. **(E)** H9N2-specific antibodies in serum that treated with RDE were determined by hemagglutination inhibition assay. The data shown were the represent of four independent experiments. **(F)** Representative histological analysis, immunohistological stainings for human CD4 T, B, and γδ-T cells in formalin-fixed paraffin-embedded spleen in humanized mice constructed with whole PBMCs after UV-H9N2 virus immunization. Scale bars = 100 μm. Bottom panels showed the higher-magnification views of the respective boxed areas in the top panels. Scale bars = 50 μm. The data shown are the mean ± SEM. *p < 0.05; **p < 0.01; ***p < 0.001. NS, no significant difference.

To further confirm the above results *in vivo*, humanized mice reconstituted with whole PBMC and certain immune cell-depleted PBMC were generated as we described before (13, 32). The humanized mice were immunized with UV-inactivated H9N2 virus, and serum total and antigen-specific Abs were detected. As shown in Figures 6A,B, serum total IgM and IgG levels in CD4/Vγ9Vδ2 T cell-deficient humanized mice were significantly lower than that in CD4 T cell-deficient humanized mice. By contrast, the serum total IgM and IgG showed no significant decrease in Vγ9Vδ2 T cell-deficient humanized mice compared with that in humanized mice reconstituted with whole PBMC. Compared with total IgG and IgM, similar results were also found for serum H9N2 virus-specific IgM, IgG, and HAI titers in humanized mice (Figures 6C–E). However, different from total Abs, serum virus-specific IgM and IgG and HAI titers in Vγ9Vδ2 T cell-deficient humanized mice were significantly reduced compared with that in humanized mice reconstituted with whole PBMC (Figures 6C–E). Therefore, our data demonstrated in humanized mice that even Vγ9Vδ2-T cells were unable to improve virus-specific antibody productions by themselves, they could induce total IgG and IgM production and facilitate virus-specific antibody productions in a CD4 T cell-dependent manner.

To identify whether CD4 T cells, B cells, and γδ-T cells directly contact *in vivo*, we stained and visualized these cells on a single paraffin-embedded slide of the spleen from humanized mice reconstituted with whole PBMC. The double staining showed the localizations of human CD4^+^-CD20^+^, CD4^+^-γδ-TCR^+^, and CD20^+^-γδ-TCR^+^ cells (Figure 6F), which verified the direct contact of these cells in spleen of humanized mice.

## Discussion

In this study, we demonstrated that Vγ9Vδ2-T cells could increase H9N2 influenza virus-specific IgG and IgM productions in a CD4 T cell dependent manner *in vitro*. Furthermore, using humanized mice, we confirmed that Vγ9Vδ2-T cells could facilitate influenza virus-specific Abs production in a CD4 T cell dependent manner *in vivo*. This, to the best of our knowledge, is the first report to show that human γδ-T cells could facilitate antigen-specific antibody production, PC differentiation, as well as Ig isotype switching. We further found that Vγ9Vδ2-T cells had synergizing effects with CD4 T cells on promoting the differentiations of CD4^+^ Tfh cells and CD19^+^IgD^−^CD38^++^ PCs, and improving B cell Ig isotype switching. During this process, Vγ9Vδ2-T cells acquired Tfh-associated features especially in the presence of CD4 T cells, indicating a reciprocal effect between Vγ9Vδ2-T and CD4 T cells during the differentiation of Tfh-like cells.

It is well accepted that Tfh cell differentiation is a multistep process including initial activation by DCs within the T cell zone, and followed by conjugations with B cells at the T-B border or within the follicle (6, 39). Previous *in vivo* study had shown that the interaction between T and B cells is crucial for Tfh cell differentiation and other non-B cells with antigen-presenting ability could also replace B cells to help CD4^+^ Tfh cell differentiation (40). Vγ9Vδ2-T cells have an unexpected role in the initiation of the adaptive immune process, as they display characteristics of professional APCs that efficiently process and present antigens to naïve αβ T cells (41). Here, we found that cell–cell contact between CD4 T and Vγ9Vδ2-T cells was crucial for CD4^+^ Tfh cell generation, and Vγ9Vδ2-T cells exhibited high CD86, CD80, and MHCII expression during influenza virus stimulation *in vitro* (data not shown here). In the spleen of humanized mice reconstituted with whole PBMCs, we further observed the co-localization of CD20^+^ B cells, CD4 T cells, and Vγ9Vδ2-T cells. Thus, we believe that these APC-like Vγ9Vδ2-T cells present antigen to CD4 cells and support CD4^+^ Tfh cell differentiation as well as proliferation in a cell–cell contact-dependent manner.

Previous *in vitro* studies have shown that human IL-6, IL-12, and IL-21 are involved in promoting the commitment of naïve CD4^+^ T cells into the Tfh lineage (9, 42, 43). Both human IL-6 and IL-12 have been demonstrated to induce IL-21 production in human studies (42). More recently, it was reported that human IL-21 was important for Vγ9Vδ2-T cells to acquire Tfh-associated features (22, 44). However, whether Vγ9Vδ2-T cells contribute to these cytokines production remains unknown. In this study, we found that Vγ9Vδ2-T cells could further increase the productions of IL-6, IL-21, and IL-13. Besides IL-6 and IL-21 that have been shown to promote Tfh cell differentiation (27), we demonstrated that IL-13 was also involved in inducing and polarizing the differentiations of both Tfh-like Vγ9Vδ2-T and CD4^+^ Tfh cells. Furthermore, our study showed at the first time that Vγ9Vδ2- and CD4 T cells could help each other to differentiate into Tfh cells, indicating a reciprocal effect between Vγ9Vδ2-T and CD4 T cells in the differentiation of Tfh-like cells.

Upon exposure to appropriate stimuli, B cells will undergo an iterative process of proliferation, somatic mutation of rearranged Ig genes. Some fraction of these proliferating B cells will secrete Abs and are referred to as plasmablasts (45–47). Both ligation of CD40 and a second helper signal provided by cytokines have been demonstrated to induce B cells isotype switching and proliferation in response to T cell-dependent signals (24). However, whether Vγ9Vδ2-T cells participate in B cell division and PC differentiation is still unknown. In this study, we identified a greater degree of proliferation of B cells in the presence of both CD4 T and Vγ9Vδ2-T cells, and almost all the proliferating Ki67^+^ B cells expressed IgG, indicating that the IgG^+^ B cells had undergone the greatest number of division. Here, we also noted significantly upregulated IL-13 in the presence of both CD4 T cells and Vγ9Vδ2-T cells compared with CD4 T cells alone. Like IL-4, IL-13 was found to be secreted by CD4^+^ Tfh cells and responsible for B cell proliferation and total IgM, IgG1 antibody production (30, 48, 49). In support of this, we showed that blocking IL-13 significantly reduced the frequency of CD19^+^Ki67^+^IgG^+^ cells and the number of CD19^+^IgD^−^CD38^++^ PCs. Therefore, for the first time, here we demonstrated that Vγ9Vδ2-T cells enabled CD4 T cells to secret more IL-13, and subsequently promoted B cells to entry into cell division, isotype switching, and differentiate into a rapidly dividing IgG-secreting cells.

B cells can process and present antigen in association with MHC class II molecules, thereby recruiting antigen-specific CD4 T cell help and stimulating B cell proliferation and differentiation (50, 51). Thus, generation of antigen-specific antibody requires cooperation between antigen-specific T and B lymphocytes. Structure analysis of γδ TCR showed several Ig-like characteristics. The Ig-like features of γδ TCR indicated that these cells could interact with naïve compounds, not with MHC-related antigens (52, 53). Indeed, here we showed a reciprocal effect of Vγ9Vδ2-T cells and CD4 T cells on enhancing humoral response against influenza virus stimulation, but Vγ9Vδ2-T cell alone could not support antigen-specific antibody production. Therefore, we believe that Vγ9Vδ2-T cells provide general help to B cells and CD4^+^ Tfh cells, and then enhance humoral immune responses, while MHC-restricted CD4 T cells drive antigen specificity by regulating affinity maturation and clone selection.

In conclusion, our study demonstrated that human γδ-T cells could facilitate influenza virus-specific antibody production, PC differentiation, as well as Ig isotype switching. This study gives a greater scope for Vγ9Vδ2-T cells in adaptive immunity, especially for the Tfh development and humoral immune responses.

## Ethics Statement

Human PBMC were collected from the buffy coats, which were obtained from the Hong Kong Red Cross approved by the Institutional Review Board of the University of Hong Kong/Hospital Authority Hong Kong West Cluster (IRB number: UW 07-154). All animal studies were approved and performed in compliance with the guidelines for the use of experimental animals by the Committee on the Use of Live Animals in the Teaching and Research (CULATR), the University of Hong Kong (CULATR number: 2986-13).

## Author Contributions

WT initiated the project, designed the experiments, and wrote the manuscript. QC, AL, ML, and KW designed and performed the experiments. KW, KN, and ZX helped accomplish some of the animal experiments. KW, YL, and WT edited the manuscript.

## Conflict of Interest Statement

The authors declare that the research was conducted in the absence of any commercial or financial relationships that could be construed as a potential conflict of interest.

## Supplementary Material

Figure S1 **(A,B)** The total number of B cells and CD4 T cells were recorded on day 7. **(C–F)** Fresh isolated Vγ9Vδ2-T cells were cultured with naïve CD4 T cells, then, the pre-activated Vγ9Vδ2-T cells (A.γδT) were added in different groups. Total IgG and IgM in supernatant on day 7 were detected by enzyme-linked immunosorbent assay (ELISA) **(C,D)**. H9N2 virus-specific IgG and IgM were detected by ELISA on day 7 **(E,F)**. **(G–J)** Intracellular IL-13, IL-21, and IL-6 in fresh Vγ9Vδ2-T cells and pre-activated (A.γδT) were detected by stimulating cells with PMA, inomycin, and BFA within the last 6 h. The each dot means one donor. The data shown are the mean ± SEM. **p* < 0.05. NS, no significant difference.

Figure S2 **(A)** Number of CD40L^+^CD4^+^ T cells was detected at day 3 after coculture. **(B,C)** Percentage of OX40^+^CD4^+^ T cells and ICOS^+^CD4^+^ T cells were detected on day 5 after coculture. **(D–F)** Supernatant were collected on day 5 and detected by Cytometric Bead Array. Data showed the productions of IL-10 **(D)**, TNF-α **(E)**, and IL-12 **(F)**. **(G,H)** Percentage of IL-13^+^Vγ9Vδ2-T cells and IL-21^+^Vγ9Vδ2-T cells were detected on day 5. **(I)** The number of PD1^+^CXCR5^+^Vγ9Vδ2-T cells was recorded after adding IL-13 neutralization antibody on day 5. **(J)** The number of CD19^+^IgD^−^CD38^++^ plasma cells was recorded after adding IL-13 or IL-21 neutralization antibodies or both of them on day 6. **p* < 0.05; ***p* < 0.01. NS, no significant difference.
