# Supplementary figures and images for: Human Vγ9Vδ2-T Cells Synergize CD4+ T Follicular Helper Cells to Produce Influenza Virus-Specific Antibody
Source: Front Immunol. 2018 Apr 4;9:599. doi: 10.3389/fimmu.2018.00599 (PMC5893649; doi:10.3389/fimmu.2018.00599)

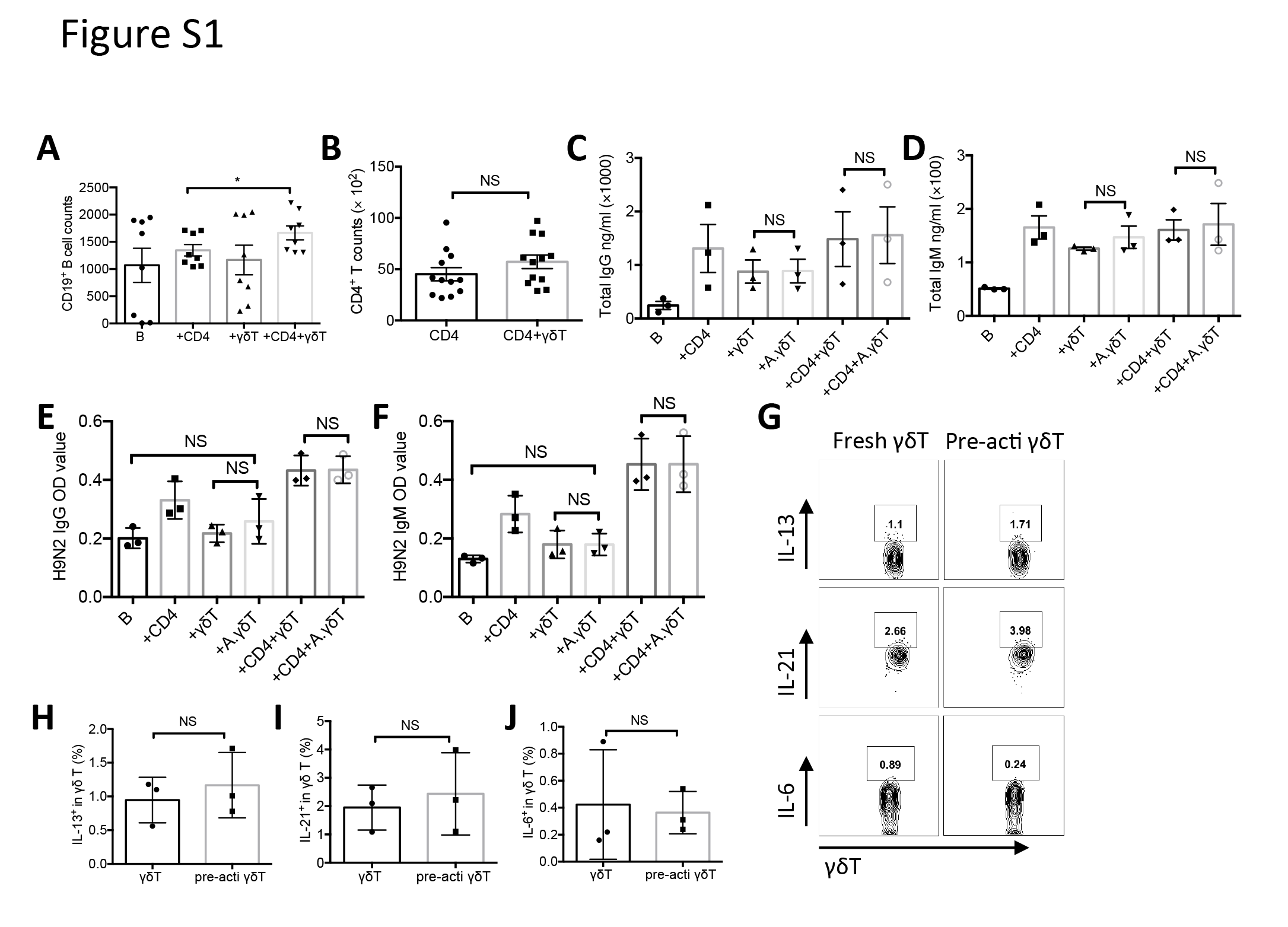

Supplement: Figure S1 — (A,B) The total number of B cells and CD4 T cells were recorded on day 7. (C–F) Fresh isolated Vγ9Vδ2-T cells were cultured with naïve CD4 T cells, then, the pre-activated Vγ9Vδ2-T cells (A.γδT) were added in different groups. Total IgG and IgM in supernatant on day 7 were detected by enzyme-linked immunosorbent assay (ELISA) (C,D). H9N2 virus-specific IgG and IgM were detected by ELISA on day 7 (E,F). (G–J) Intracellular IL-13, IL-21, and IL-6 in fresh Vγ9Vδ2-T cells and pre-activated (A.γδT) were detected by stimulating cells with PMA, inomycin, and BFA within the last 6 h. The each dot means one donor. The data shown are the mean ± SEM. *p < 0.05. NS, no significant difference. [file image_1.tif]

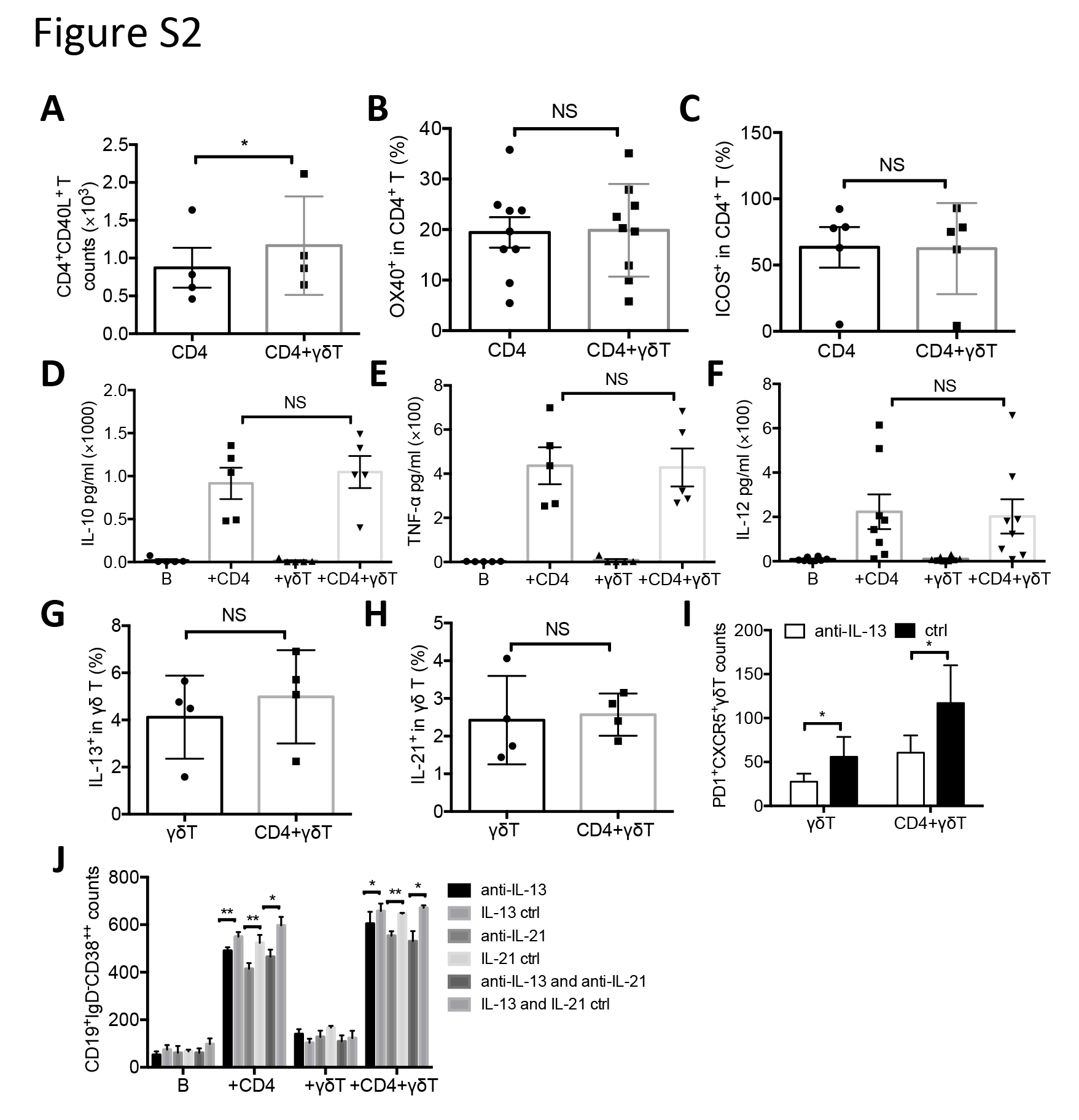

Supplement: Figure S2 — (A) Number of CD40L+CD4+ T cells was detected at day 3 after coculture. (B,C) Percentage of OX40+CD4+ T cells and ICOS+CD4+ T cells were detected on day 5 after coculture. (D–F) Supernatant were collected on day 5 and detected by Cytometric Bead Array. Data showed the productions of IL-10 (D), TNF-α (E), and IL-12 (F). (G,H) Percentage of IL-13+Vγ9Vδ2-T cells and IL-21+Vγ9Vδ2-T cells were detected on day 5. (I) The number of PD1+CXCR5+Vγ9Vδ2-T cells was recorded after adding IL-13 neutralization antibody on day 5. (J) The number of CD19+IgD−CD38++ plasma cells was recorded after adding IL-13 or IL-21 neutralization antibodies or both of them on day 6. *p < 0.05; **p < 0.01. NS, no significant difference. [file image_2.tif]
